# SARS-CoV-2 infection in chronic kidney disease patients with pre-existing dialysis: description across different pandemic intervals and effect on disease course (mortality)

**DOI:** 10.1007/s15010-022-01826-7

**Published:** 2022-04-29

**Authors:** Lisa Pilgram, Lukas Eberwein, Bjoern-Erik O. Jensen, Carolin E. M. Jakob, Felix C. Koehler, Martin Hower, Jan T. Kielstein, Melanie Stecher, Bernd Hohenstein, Fabian Prasser, Timm Westhoff, Susana M. Nunes de Miranda, Maria J. G. T. Vehreschild, Julia Lanznaster, Sebastian Dolff, the LEOSS study group, Julia Lanznaster, Bjoern-Erik Jensen, Martin Hower, Bernd Hohenstein, Timm Westhoff, Maria Vehreschild, Christoph Spinner, Maria Madeleine Ruethrich, Lukas Tometten, Stefan Borgmann, Norma Jung, Bernd Hertenstein, Christian Degenhardt, Ingo Voigt, Frank Hanses, Kai Wille, Juergen vom Dahl, Katja Rothfuss, Kerstin Hellwig, Jan Rupp, Nora Isberner, Lukas Eberwein, Jacob Nattermann, Richard Strauss, Sebastian Dolff, Siri Göpel, Jörg Janne Vehreschild, Susana M. Nunes de Miranda, Carolin E. M. Jakob, Melanie Stecher, Lisa Pilgram, Nick Schulze, Sandra Fuhrmann, Max Schons, Annika Claßen, Bernd Franke, Fabian Prasser

**Affiliations:** 1 Department of Nephrology and Medical Intensive Care, Charité, Universitätsmedizin Berlin, Berlin, Germany; 2 Department of Internal Medicine, Hematology and Oncology, Goethe University Frankfurt, Frankfurt, Germany; 3 4th Department of Internal Medicine, Klinikum Leverkusen gGmbH, Leverkusen, Germany; 4 Department of Gastroenterology, Hepatology and Infectious Diseases, Heinrich Heine University, Düsseldorf, Germany; 5 Department I of Internal Medicine, Faculty of Medicine and University Hospital Cologne, University of Cologne, Cologne, Germany; 6 German Centre for Infection Research (DZIF), Partner Site Bonn-Cologne, Cologne, Germany; 7 Department II of Internal Medicine and Center for Molecular Medicine Cologne, Faculty of Medicine and University Hospital Cologne, University of Cologne, Cologne, Germany; 8 Faculty of Medicine and University Hospital Cologne, CECAD, University of Cologne, Cologne, Germany; 9 Department of Pneumology, Infectiology, Internal Medicine and Intensive Care, Klinikum Dortmund gGmbH, Dortmund, Hospital of University Witten/Herdecke, Dortmund, Germany; 10 Medical Clinic V, Nephrology|Rheumatology|Blood Purification, Academic Teaching Hospital Braunschweig, Braunschweig, Germany; 11 Nephrological Centre Villingen-Schwenningen, Villingen-Schwenningen, Germany; 12 Berlin Institute of Health at Charité - Universitätsmedizin Berlin, Berlin, Germany; 13 Department of Internal Medicine I, Marien Hospital Herne Ruhr University Bochum, Herne, Germany; 14 Department of Internal Medicine, Infectious Diseases, Goethe University Frankfurt, Frankfurt, Germany; 15 Department of Internal Medicine 2, Klinikum Passau, Passau, Germany; 16 Department of Infectious Diseases, West German Centre of Infectious Diseases, University Hospital Essen, University Duisburg-Essen, Hufelandstr. 55, 45122 Essen, Germany

**Keywords:** COVID-19, Hemodialysis, CKD5D, Kidney, SARS-CoV-2

## Abstract

**Purpose:**

Patients suffering from chronic kidney disease (CKD) are in general at high risk for severe coronavirus disease (COVID-19) but dialysis-dependency (CKD5D) is poorly understood. We aimed to describe CKD5D patients in the different intervals of the pandemic and to evaluate pre-existing dialysis dependency as a potential risk factor for mortality.

**Methods:**

In this multicentre cohort study, data from German study sites of the Lean European Open Survey on SARS-CoV-2-infected patients (LEOSS) were used. We multiply imputed missing data, performed subsequent analyses in each of the imputed data sets and pooled the results. Cases (CKD5D) and controls (CKD not requiring dialysis) were matched 1:1 by propensity-scoring. Effects on fatal outcome were calculated by multivariable logistic regression.

**Results:**

The cohort consisted of 207 patients suffering from CKD5D and 964 potential controls. Multivariable regression of the whole cohort identified age (> 85 years adjusted odds ratio (aOR) 7.34, 95% CI 2.45–21.99), chronic heart failure (aOR 1.67, 95% CI 1.25–2.23), coronary artery disease (aOR 1.41, 95% CI 1.05–1.89) and active oncological disease (aOR 1.73, 95% CI 1.07–2.80) as risk factors for fatal outcome. Dialysis-dependency was not associated with a fatal outcome—neither in this analysis (aOR 1.08, 95% CI 0.75–1.54) nor in the conditional multivariable regression after matching (aOR 1.34, 95% CI 0.70–2.59).

**Conclusions:**

In the present multicentre German cohort, dialysis dependency is not linked to fatal outcome in SARS-CoV-2-infected CKD patients. However, the mortality rate of 26% demonstrates that CKD patients are an extreme vulnerable population, irrespective of pre-existing dialysis-dependency.

**Supplementary Information:**

The online version contains supplementary material available at 10.1007/s15010-022-01826-7.

## Introduction

Several hundred million people were infected and more than 5 million people died since the beginning of the coronavirus disease 2019 (COVID-19) pandemic [1]. COVID-19 as a respiratory syndrome caused by the infection with severe acute respiratory syndrome coronavirus 2 (SARS-CoV-2) and it is characterized by fever, cough and dyspnea with a broad clinical spectrum ranging from lack of symptoms to death. COVID-19 pneumonia is a well-known and frequent organ manifestation in patients with severe disease. SARS-CoV-2 interacts with the transmembrane protein angiotensin converting enzyme 2 (ACE-2), best known for its role in the renin–angiotensin–aldosterone system (RAAS). ACE-2 is expressed in alveolar cells in the lung, as well as in the kidney, most abundant in proximal tubular cells and podocytes [2]. There is increasing evidence that the kidney is a target organ as well [3, 4]. In line, SARS-CoV-2 RNA can be detected in 60% of kidney specimens of COVID-19 patients suggesting renal tropism and a pivotal role in the pathogenesis [4].

Apart from being a target of the virus itself, pre-existing chronic kidney disease (CKD) has been reported to be both, a risk factor for a more severe course of the disease as well as mortality [5, 6]. This is best epitomized in CKD5D patients adjusted for age and other comorbidities, such as atherosclerotic cardiovascular disease or chronic heart disease [7]. In a previous study, we detected a mortality higher than 30% in these patients but were not able to confirm dialysis as an independent risk factor [8]. However, data at this time was limited and only included 75 patients on dialysis. Results from the European ERA–EDTA Registry presented a COVID-19 attributable mortality of 20.0% among patients undergoing chronic dialysis [9]. In Germany similar numbers could be obtained among dialysis-dependent CKD patients [10]. However, studies including both dialysis-dependent as well as dialysis-independent CKD patients are scarce which might underestimate the risk of dialysis-independent CKD itself.

Unfortunately, therapeutic options in COVID-19 are still limited. Especially in the first interval of the pandemic, the European Medicines Agency (EMA) issued warnings for severe kidney impairment (eGFR < 30 ml/min or dialysis or veno-venous hemofiltration) in the administration of remdesivir, the only authorized drug at this time [11]. The advent of other pharmacological interventions and the changing view on remdesivir might have an impact in later intervals of the pandemic [12].

The goal of the present study was to describe the course of SARS-CoV-2 infection in patients suffering from dialysis-dependent CKD across the pandemic intervals and to evaluate the influence of pre-existing dialysis based on data from the Lean European Open Survey on SARS-CoV-2-infected patients (LEOSS).

## Methods

### Study design and data collection

**Fig. 1:**
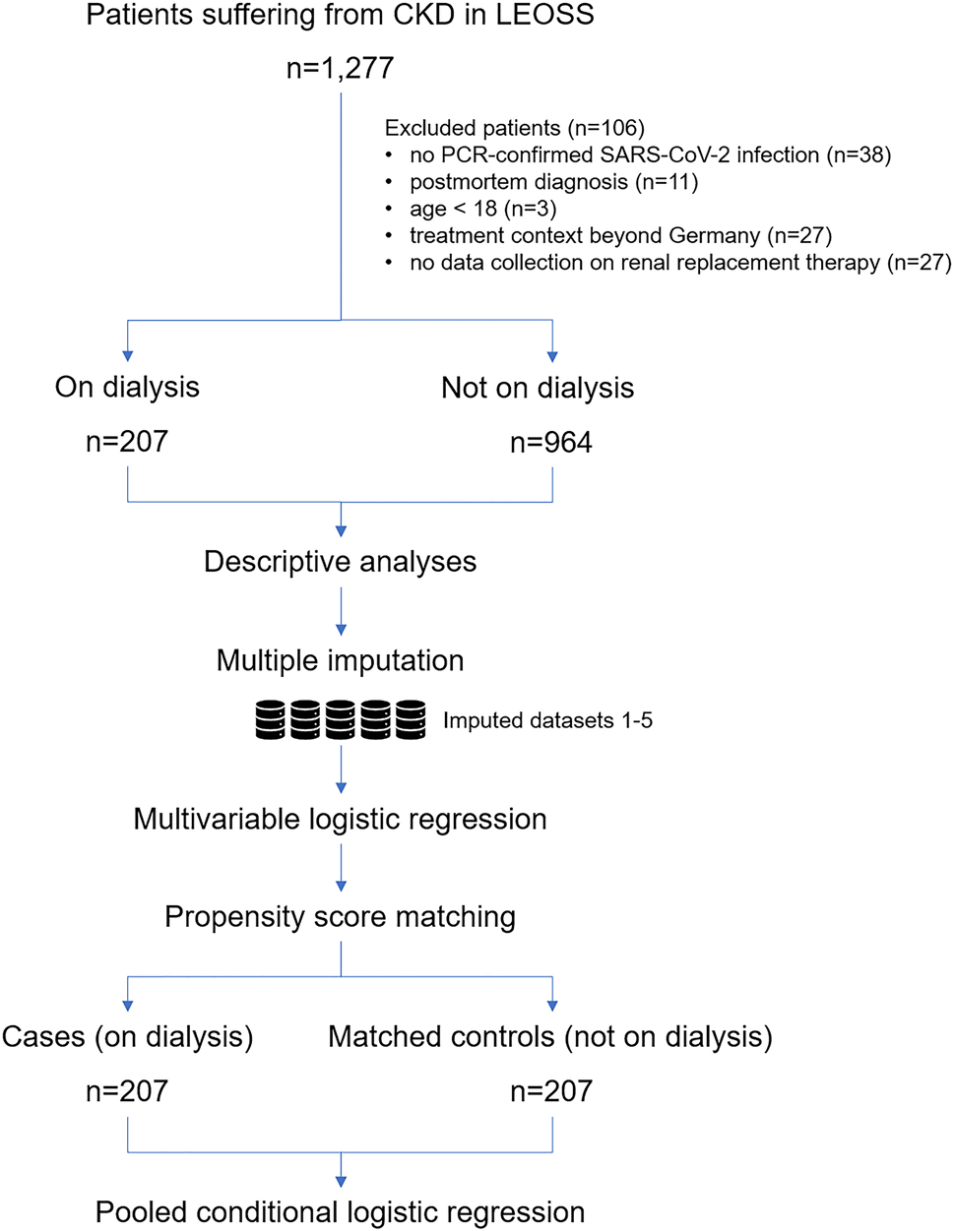
Study flow chart. We extracted patients suffering from CKD from LEOSS and applied the indicated exclusion criteria. Patients on dialysis were described and compared throughout the different phases of pandemic using the original data set. Missing values were multiply imputed. Missing analyses are displayed in Table S1. Each case of each imputed data set was matched via propensity score matching to one control. The latter was defined as patients suffering from CKD not requiring dialysis. Results of the conditional logistic regression stratified by dialysis were pooled across the 5 imputed data sets. CKD: chronic kidney disease. LEOSS: Lean European Open Survey on SARS-CoV-2-infected patients

We performed our analyses of patients suffering from dialysis-dependent CKD retrieving data from LEOSS (https://leoss.net/) (Fig. 1) [13]. In LEOSS, clinical data is reported anonymously and retrospectively in an electronic case report form using the online platform ClinicalSurveys.net of the University Hospital of Cologne [14]. The anonymization procedure has been published previously [15].

### Study population

The transmitted data set consisted of 1277 patients, which were documented in 80 different study sites and diagnosed between January 2020 and May 2021. Exclusion criteria are illustrated in Fig. 1. We excluded in total 8.3% (106/1277) patients resulting in a data set of 207 patients suffering from CKD5D (cases) and 964 patients suffering from CKD not requiring dialysis (potential controls).

### Covariables and outcomes

Chosen parameters included sociodemographics, comorbidities, details on CKD, clinical and diagnostic parameters, as well as administered therapies. Symptoms and diagnostic parameters were determined within 48 h after first SARS-CoV-2 positive testing. Pre-existing comorbidities and clinical events were documented by investigators according to clinical definitions using anamnestic information and medical records. Prior immunosuppressive medication at baseline was defined as administered within an interval of 3 months before SARS-CoV-2 infection. Advanced respiratory support was defined as mechanical ventilation or extracorporeal membrane oxygenation (ECMO). The month of first diagnosis was assigned to one of three intervals of pandemic based on infection rates and evolving virus variants in Germany [16]: January 2020–June 2020, July 2020–January 2021, February 2021–May 2021. Death within the observational period was used as end-point in the regression analyses.

### Statistical methods

Data management and analyses were performed using R, version 4.1.0 [17]. Figure 1 illustrates the workflow.

We described patients’ characteristics as absolute numbers and percentages. Group differences between the three different intervals of pandemic were determined using Chi-squared test or when applicable Fisher’s exact test. We controlled for multiple comparison using the Bonferroni correction.

Variables relevant for the regression analyses were analysed for missingness (supplementary (S) Table S1) and imputed iteratively via fully conditional specification (FCS) with proportional odds model or polytomous logistic regression depending on the nature of the respective variable using the R package MICE (https://cran.rproject.org/web/packages/mice/mice.pdf). This resulted in a total of 5 imputed data sets.

For the propensity-score matched pair analysis, each case was matched to one control in each imputed data set using the R package MatchIt (https://cran.rproject.org/web/packages/MatchIt/index.html). Exact matching was performed on age, gender and phase (according to LEOSS criteria, see Figure S1) at first SARS-CoV-2 detection; propensity-score matching (nearest neighbour) on hypertension, chronic heart failure, coronary artery disease, diabetes mellitus type 2, chronic obstructive pulmonary disease (COPD), active oncological disease, obesity, prior immunosuppressive medication and therapy limitations. Matching quality was assessed via standardized mean differences.

We used multivariable logistic regression or conditional logistic regression stratified by dialysis to estimate effects. Results were pooled across all imputed data sets and reported as (adjusted) odds ratios [(a)OR] with 95% confidence intervals (95% CI). *p* < 0.05 was set as level of significance.

### Ethical statement

LEOSS was approved by the applicable local ethics committees of all participating centers and registered at the German Clinical Trials Register (DRKS, No. S00021145).

## Results

### Cohort

The cohort consisted of 207 patients suffering from CKD5D recruited in LEOSS and diagnosed between January 2020 and May 2021. All patients underwent hemodialysis. Vascular hypertensive (46.2%, 61/132), secondary (22.0%, 29/132) and primary glomerular disease (9.1%, 12/132) were the leading etiologies of CKD. Most CKD5D patients also suffered from hypertension (79.6%, 164/206). Other frequent comorbidities included diabetes mellitus type 2 (44.2%, 88/199), coronary artery disease (36.3%, 74/204) and obesity (31.4%, 44/140). Prior immunosuppressive medication was present in 17.6% (34/193). History of transplantation (51.5%, 17/33), rheumatological disease (15.2%, 5/33) and other reasons (33.3%, 11/33) were given as indication. Details are depicted in Table 1.

**Table 1:** Characteristics of SARS-CoV-2-infected patients on hemodialysis in the different intervals of COVID-19 pandemic

	Diagnosed between						*p*-value
	January 2020 and June 2020		July 2020 and January 2021		February 2021 and May 2021		
	*n* = 58	%	*n* = 116	%	*n* = 33	%	
Age							
18–45 years	4/58	6.9	8/116	6.9	3/33	9.1	0.751
46–55 years	3/58	5.2	12/116	10.3	4/33	12.1	
56–65 years	10/58	17.2	25/116	21.6	7/33	21.2	
66–75 years	12/58	20.7	22/116	19.0	8/33	24.3	
76–85 years	21/58	36.2	41/116	35.3	7/33	21.2	
> 85 years	8/58	13.8	8/116	6.9	4/33	12.1	
Gender							
Female	20/58	34.5	47/116	40.5	20/33	60.6	0.046
Male	38/58	65.5	69/116	59.5	13/33	39.4	
Comorbidities							
Hypertension	44/58	75.9	96/115	83.5	24/33	72.7	0.283
Chronic heart failure	16/56	28.6	26/111	23.4	11/33	33.3	0.483
Coronary artery disease	20/57	35.1	43/114	37.7	11/33	33.3	0.878
Diabetes mellitus type 2	19/56	33.9	54/111	48.7	15/32	46.9	0.185
COPD	5/58	8.6	13/112	11.6	5/33	15.2	0.603
Active oncological disease	2/58	3.5	4/105	3.8	1/33	3.0	1.000
Obesity	13/52	25.0	25/67	37.3	6/21	28.6	0.341
Prior immunosuppressive medication							
Prior immunosuppressive medication	7/56	12.5	18/107	16.8	9/30	30	0.121
Status at COVID-19 diagnosis							
Uncomplicated phase	39/58	67.2	76/116	65.5	18/33	54.6	0.432
Complicated phase	17/58	29.3	37/116	31.9	12/33	36.4	
Critical phase	2/58	3.5	3/116	2.6	3/33	9.1	
Treatment in the course							
Steroids	4/52	7.7	48/111	43.2	10/33	30.3	< 0.001
Remdesivir	1/51	2.0	9/107	8.4	1/33	3.0	0.239
Convalescent plasma	1/41	2.4	7/109	6.4	1/23	4.4	0.785
Targeted therapy (antibodies)	NA	NA	0/72	0.0	4/19	21.1	< 0.001
Apheresis	2/42	4.8	1/106	0.9	0/23	0.0	0.204
Chloroquin	8/51	15.7	2/106	1.9	1/33	3.0	0.004
Azithromycin	7/52	13.5	7/108	6.5	2/33	6.1	0.234
Therapy limitation							
Explicit deny of therapy	5/24	20.8	26/109	23.9	5/21	23.8	0.718
Explicit wish for therapy	1/24	4.2	2/109	1.9	1/21	4.8	
No discussion on therapy limitations	18/24	75.0	81/109	74.3	15/21	71.4	
Course of disease							
Fatal outcome	15/58	25.9	28/116	24.1	12/33	36.4	0.370
Advanced respiratory support	11/54	20.4	14/115	12.2	4/33	12.1	0.345
Critical phase	17/58	29.3	18/116	15.5	6/33	18.2	0.096
Thrombotic event	2/44	4.6	3/114	2.6	2/23	8.0	0.317
Bleeding event	0/39	0.0	4/113	3.5	0/25	0.0	0.760
Septic shock	3/56	5.4	6/116	5.2	0/33	0.0	0.467
Congestive heart failure	0/56	0.0	1/115	0.9	1/33	0.3	0.374

All variables are derived from the unimputed data set and expressed as numbers (no.) and percentages (%) referred to the numbers excluding missing data (missing details in Table S1). Obesity was defined by an indicated Body-Mass-Index > 30 kg/m^2^. Prior immunosuppressive medication includes an interval of 3 months before SARS-CoV-2 infection, therapy limitation defined as Do-Not-Intubate-, Do-Not-Resuscitate-Orders or the refusal of intensive care, advanced respiratory support as invasive or non-invasive mechanical ventilation or ECMO. COPD: chronic obstructive pulmonary disease. ECMO: extracorporeal membrane oxygenation

At first detection of SARS-CoV-2, 46.2% (84/180) of our patients reported fever, 36.0% (64/178) dyspnea, 28.5% (49/172) dry cough, 7.1% (12/170) myalgia, 4.1% (7/171) headache and 2.4% (4/167) hypogeusia and/or hyposmia. At this time point, most patients (64.3%, 133/207) were assigned to the uncomplicated phase according to LEOSS criteria (see Figure S1). During the further course of disease, 19.8% (41/207) underwent critical phase, 14.3% (29/209) required advanced respiratory support and 26.6% (55/207) patients deceased.

### Patients’ characteristics, presentation at first SARS-CoV-2 detection and treatment strategies in different pandemic intervals

The pandemic was divided into three intervals based on infection rates and evolving virus variants in Germany: January 2020–June 2020, July 2020–January 2021, February 2021–May 2021. When comparing across the pandemic intervals, patients’ characteristics did not differ except for gender (Table 1). The percentage of recruited female patients increased over time: in the first interval, 34.5% (20/58) of the patients, in the second 40.5% (47/116) and in the third interval 60.6% (20/33).

**Fig. 2:**
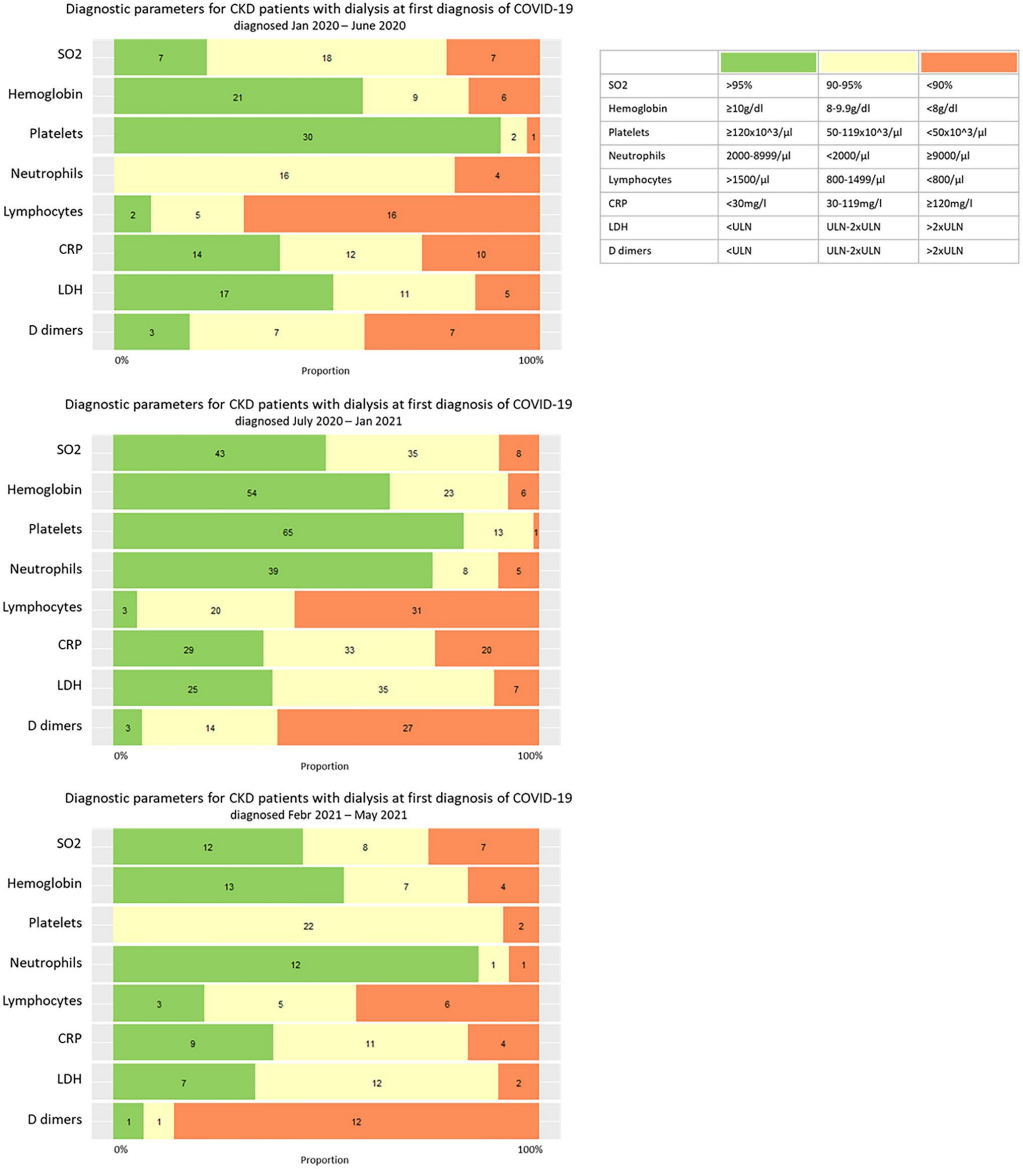
Diagnostic parameters for SARS-CoV-2-infected patients on hemodialysis at first diagnosis of COVID-19 in the different intervals of COVID-19 pandemic. Proportion referred to the numbers excluding missing data (missing details in Table S1) and numbers in the specified categories of the indicated diagnostic parameters are displayed using the unimputed data set. The diagnostic parameters were determined closest to the first diagnosis but did not exceed 48 h after SARS-CoV-2 positive testing. Timing of first diagnosis was aggregated into three intervals of pandemic based on the epidemiological waves in Germany: January 2020–June 2020, July 2020–January 2021 and February 2021–May 2021; diagnostic assessment into three categories as defined in the legend. CKD: chronic kidney disease. SO_2_: oxygen saturation in arterial blood. CRP: C-reactive protein. LDH: lactate dehydrogenase. ULN: upper limit of normal in the respective local laboratory

There was no significant difference between the pandemic intervals regarding existing symptoms and laboratory parameters at first SARS-CoV-2 detection. The latter is illustrated in Fig. 2. CRP was generally elevated ≥ 30 mg/dl in 63.4% (90/142), D-dimers > 2 × upper limit of normal (ULN) in 61.3% (46/75) and lymphocytes were below 800/μl in 58.2% (52/91).

Therapy limitations were reported in 23.4% (36/154) of the patients. Throughout the pandemic, administered treatment changed: Steroids > 0.5 mg/kg prednisolone equivalents were used in 7.7% (4/52) of the patients during the first interval, in 43.2% (48/111) during the second one and in 30.3% (10/33) during the third interval. Use of chloroquine was more frequent in the first interval (15.7%, 8/51 versus 1.9%, 2/106 in the second interval versus 3.0%, 1/33 in the third interval). Remdesivir was administered in 5.8% (11/191) of the patients with no differences in frequency of use throughout the pandemic.

### Estimating the effect of dialysis in CKD patients

We performed a multivariable logistic regression on fatal outcome using the whole data set of 1171 patients suffering from CKD in LEOSS after multiple imputation. Pooled results are shown in Table 2. Increasing age was identified as risk factor with the greatest adjusted odds ratio (aOR) in the category > 85 years (aOR 7.34, 95% CI 2.45–21.99, *p* < 0.001). Chronic heart failure (aOR 1.67, 95% CI 1.25–2.23, *p* < 0.001), coronary artery disease (aOR 1.41, 95% CI 1.05–1.89, *p* = 0.021) and active oncological disease (aOR 1.73, 95% CI 1.07–2.80, *p* = 0.027) were further predictors for fatal outcome. Dialysis dependency did not show a significant association with mortality in CKD patients (aOR 1.08, 95% CI 0.75–1.54, *p* = 0.692).

**Table 2:** Pooled results of multivariable logistic regression of predictive factors for fatal outcome in SARS-CoV-2-infected patients suffering from chronic kidney disease

	Multivariable regression analysis on fatal outcome			
	aOR	95% CI		*p*-value
Age				
18–45 years	Reference			
46–55 years	3.01	0.95	9.60	0.062
56–65 years	1.72	0.56	5.33	0.344
66–75 years	3.08	1.04	9.13	0.043
76–85 years	3.95	1.35	11.55	0.012
> 85 years	7.34	2.45	21.99	< 0.001
Gender				
Female	0.87	0.66	1.15	0.340
Male	Reference			
Comorbidities*				
Hypertension	0.98	0.67	1.37	0.897
Chronic heart failure	1.67	1.25	2.23	< 0.001
Coronary artery disease	1.41	1.05	1.89	0.021
Diabetes mellitus type 2	0.97	0.73	1.28	0.810
COPD	1.28	0.86	1.91	0.223
Active oncological disease	1.73	1.07	2.80	0.027
Obesity	1.03	0.69	1.53	0.895
Pre-existing dialysis	1.08	0.75	1.54	0.692
Prior immunosuppressive medication*				
Prior immunosuppressive medication	0.98	0.65	1.48	0.918

Multivariable logistic regression on fatal outcome was performed using the imputed data set. Obesity was defined by an indicated Body-Mass-Index > 30 kg/m^2^. Prior immunosuppressive medication includes an interval of 3 months before SARS-CoV-2 infection, therapy limitation defined as Do-Not-Intubate-, Do-Not-Resuscitate-Orders or the refusal of intensive care, advanced respiratory support as invasive or non-invasive mechanical ventilation or ECMO. aOR: adjusted odds ratio. CI: confidence interval. COPD: chronic obstructive pulmonary disease. ECMO: extracorporeal membrane oxygenation. * No reference level indicated in binary variables

**Fig. 3:**
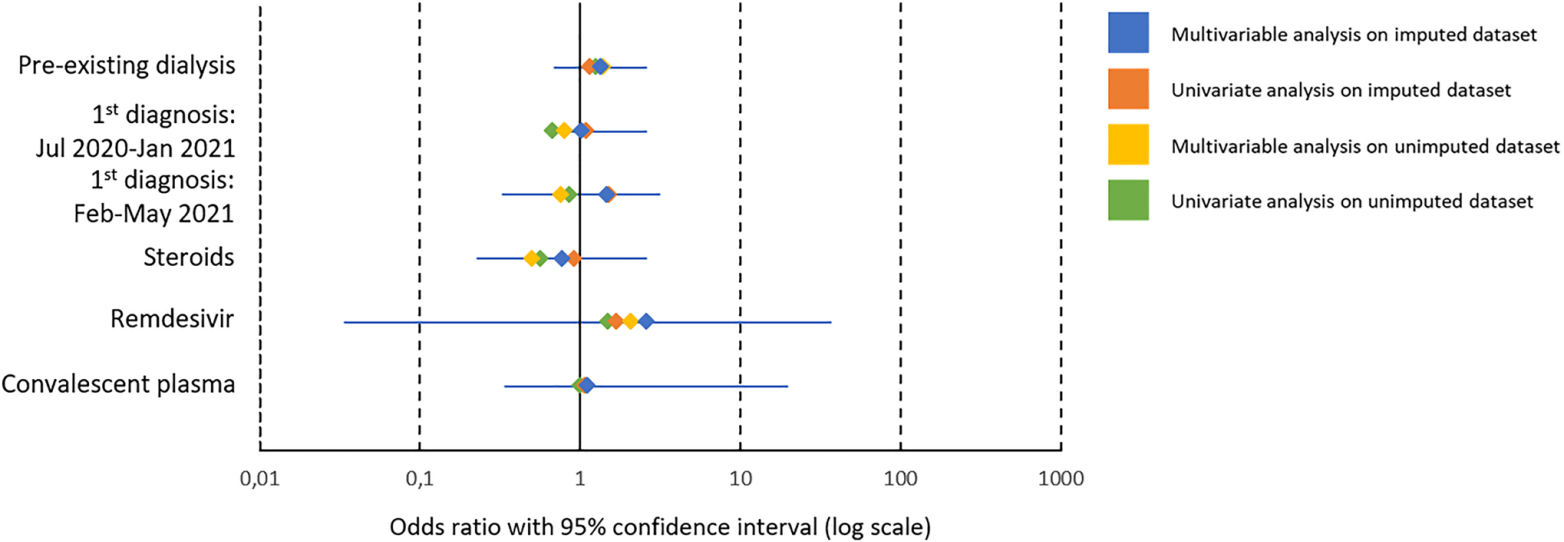
Forest plot of odds ratios from conditional regression analyses on fatal outcome stratified by dialysis. Univariate and multivariable regression analyses were performed after propensity-score matching in the imputed and unimputed data sets with the respective (adjusted) odds ratio ((a)OR) displayed in the figure. 95% confidence interval is plotted for the aOR of the multivariable conditional regression of imputed data. Timing of first diagnosis was aggregated into three intervals of pandemic based on the epidemiological waves in Germany: January 2020–June 2020 (reference category), July 2020–January 2021 and February 2021–May 2021. Treatment administered at least once in the course of COVID-19 with no administration serving as reference category

Patients suffering from CKD5D were matched via propensity score to controls suffering from CKD not requiring dialysis. Controls were predominantly assigned to CKD stage 3 (52.1%, 395/758), followed by stage 4 (15.8%, 120/758) and stage 2 (15.0%, 114/758), according to the definition of the international guideline group Kidney Disease Improving Global Outcomes (KDIGO). A detailed description of controls suffering from CKD not requiring dialysis is given in Table S2. In the univariate and multivariable conditional regression stratified by dialysis, dialysis-dependency was not significantly associated with fatal outcome (aOR 1.34, 95% CI 0.70–2.59, *p* = 0.375). Pooled results are shown in Table 3. We performed sensitivity analyses using the unimputed data set (Table S3) that confirmed our results with dialysis-dependency not being significantly associated with fatal outcome but exhibiting a risk tendency (aOR 1.40, 95% CI 0.73–2.69, *p* = 0.31). Univariate and multivariable results of the matched-pair analyses using the imputed and unimputed data set are illustrated in Fig. 3.

**Table 3:** Pooled results of conditional regression analyses on fatal outcome stratified by dialysis

	Univariate regression analysis on fatal outcome				Multivariable regression analysis on fatal outcome			
	OR	95% CI		*p*-value	aOR	95% CI		*p*-value
Pre-existing dialysis	1.15	0.66	2.01	0.617	1.34	0.70	2.59	0.375
Diagnosed between								
January–June 2020	Reference							
July 2020–January 2021	1.10	0.41	2.97	0.853	1.02	0.70	2.59	0.973
February–May 2021	1.53	0.26	9.20	0.620	1.46	0.33	3.16	0.708
Treatment in the course*								
Steroids	0.92	0.35	2.44	0.869	0.77	0.23	2.61	0.671
Remdesivir	1.69	0.34	8.43	0.515	2.61	0.03	36.41	0.342
Convalescent plasma	1.08	0.07	16.52	0.953	1.12	0.34	19.80	0.944

Univariate and multivariable regression analyses were performed after propensity-score matching, results of the imputed data sets pooled. Exact matching was performed on age, gender and phase (according to LEOSS criteria, see Figure S1) at first SARS-CoV-2 detection; propensity-score matching (nearest neighbour) on hypertension, chronic heart failure, coronary artery disease, diabetes mellitus type 2, chronic obstructive pulmonary disease (COPD), active oncological disease, obesity, prior immunosuppressive medication and therapy limitations. Timing of first diagnosis was aggregated into three intervals of pandemic based on the epidemiological waves in Germany: January 2020–June 2020 (reference category), July 2020–January 2021 and February 2021–May 2021. Treatment administered at least once in the course of COVID-19 with no administration serving as reference category. Obesity was defined by an indicated Body-Mass-Index > 30 kg/m^2^. Prior immunosuppressive medication includes an interval of 3 months before SARS-CoV-2 infection. Phases at COVID-19 diagnosis were assigned according to LEOSS criteria (Figure S1). Therapy limitation were defined as Do-Not-Intubate-, Do-Not-Resuscitate-Orders or the refusal of intensive care, advanced respiratory support as invasive or non-invasive mechanical ventilation or ECMO. (a)OR: (adjusted) odds ratio. CI: confidence interval. COPD: chronic obstructive pulmonary disease. ECMO: extracorporeal membrane oxygenation. * No reference level indicated in binary variables

## Discussion

The present study is based on data of LEOSS, which is the largest clinical data collection on SARS-CoV-2-infected patients in Germany and has been active since the very beginning of the pandemic, allowing us to describe SARS-CoV-2-infected CKD5D patients of different pandemic intervals [18]. Using a matched-pair design, we examined the additional effect of dialysis-dependency to the general risk of non-dialysis CKD patients.

### Differences across the pandemic intervals

Interestingly—despite of changing patients’ management and testing strategies, evolving virus variants and applied vaccinations—sociodemographics, clinical characteristics and laboratory findings at first diagnosis did not significantly differ in dialysis-dependent CKD patients over time. However, the changing landscape of COVID-19 therapeutics and recommendations for specific strategies, as well as the overall limited options for CKD patients is reflected in our data. While hydroxychloroquine has been more frequently used at the very beginning of the pandemic, it declined after randomized controlled trials failed to detect a benefit [19]. The increasing use of steroids follows recommendations by (inter)national medical societies and the WHO that evolved after the first interval of the pandemic [20–22]. Remdesivir, as the first antiviral drug approved but currently without clear recommendation for use [20, 22] and in particular with precaution for patients with reduced GFR [11], has interestingly been administered throughout the whole pandemic in some patients undergoing dialysis for chronic dialysis dependency.

### Risk factors in patients suffering from CKD

Our multivariable analysis confirmed already known risk factors also for patients suffering from CKD, such as age, chronic heart failure, coronary artery disease and an active oncological disease [23–25], which was described for the whole LEOSS cohort [18, 26–28] and which we published in a smaller CKD cohort previously [8]. In contrast, broadly accepted risk factors, such as male sex, hypertension or diabetes mellitus failed to present as additional risk factors in our model, which might be due to the overall high prevalence in our cohort. It might also be important to note that hypertension and diabetes mellitus often have been identified without being adjusted for CKD [23, 29] or in a cohort where prevalence of CKD was low [30, 31].

### Dialysis-dependency—an independent risk factor for mortality?

Pre-existing need for dialysis by itself was neither in our multivariable regression analysis nor in our propensity-score matched-pair analyses significantly associated with fatal outcome in SARS-CoV-2-infected patients, thus confirming our previously published results [8]. The OpenSAFELY project with 17,278,392 individuals similarly identified CKD as one of the highest risk factors for death but, in contrast, identifies a history of dialysis as an additional factor in a secondary analysis [24]. The slight difference in a history of dialysis and dialysis-dependency might account for these discrepancies. Flythe et al. addressed in a retrospective cohort study (STOP-COVID) in 4264 critical ill patients with COVID-19 (143 patients with preexisting kidney failure receiving maintenance dialysis) a similar question as the present study [5]. They demonstrated that dialysis-dependent CKD patients had a shorter interval from symptom onset to intensive care treatment than non-dialysis-dependent CKD patients and detected higher mortality rates for both—dialysis-dependent and -independent CKD patients. In line, they showed that hazards of in-hospital death is higher in patients with dialysis-dependent kidney failure compared to patients without pre-existing CKD [5]. Further studies report high mortality within the range of 20–30% among SARS-CoV-2-infected patients suffering from dialysis-dependent CKD [10, 25, 32–34] which is comparable to our results (26.6%, 55/207). A UK registry study stressed kidney replacement therapy as crucial risk factor. In particular in center hemodialysis patients had a high mortality with a peak in April 2020 [35]. A more recent prospective observational study demonstrated an increased mortality (35.7%) of hemodialysis patients within the first year after infection [36]. Remarkably, these patients died also after discharge of the hospital. Moreover, anti SARS-CoV2 antibodies decrease with time indicating that humoral responses were low after infection. A similar response has been described after vaccination in this vulnerable cohort [37]. However, in these studies, a direct comparison to dialysis-independent CKD patients is lacking. Thus, the studies analyzed different dialysis cohorts in different countries at different time point during the pandemic. Subsequently the results might differ. Our study highlights CKD and decreased glomerular filtration rate (GFR) independent of dialysis as relevant risk factors for severe COVID-19.

As 100% of our dialysis patients were on hemodialysis and none on peritoneal dialysis, we are unable to generate insights in potential benefits of hemodialysis, i.e., the intermittent anticoagulation wit heparin, usually three times a week, or the regular health care utilization that would allow swifter diagnosis and therapy. Previous reports have, however, not shown any difference in the disease course between peritoneal dialysis and hemodialysis [25].

One of the strengths of our study lies in being based on data of LEOSS, which has uniformly and standardized collected data since the beginning of the pandemic. Thus, all three intervals of the pandemic, as well as cases and controls derive from one data source operating on a transregional level with more than 131 sites. Nevertheless, there are still several limitations as outpatient sites are underrepresented in LEOSS, study sites have changed over time and important confounders (e.g., socioeconomic background, COVID-19 vaccination status, frailty) might not have sufficiently been considered in the matching or regression analysis. Dialysis could also have an impact on other endpoints, such as ICU admission or thromboembolic complications which should be addressed in further analyses.

In conclusion, our results indicate that not chronic dialysis dependency itself but rather the associated age, co-morbidities and underlying diseases are important modifiers of disease severity and death. However, the high mortality in both, cases and controls, should raise awareness for SARS-CoV-2-infected patients suffering from CKD, and should be considered when discussing about recommendations for vaccine booster shots.

## Supplementary Information

Below is the link to the electronic supplementary material.

Supplementary file1 (DOCX 101 KB)

## Data Availability

The data for the analyses of this study are retrieved from LEOSS. A public data set from LEOSS is online available (https://leoss.net/data/). Access to a more extensive data set can be requested online (https://leoss.net/statistics/) and is discussed within the governance organs.
