# Supplementary material for: SARS-CoV-2 infection in chronic kidney disease patients with pre-existing dialysis: description across different pandemic intervals and effect on disease course (mortality)
Source: Infection. 2022 Apr 29;51(1):71–81. doi: 10.1007/s15010-022-01826-7 (PMC9052729; doi:10.1007/s15010-022-01826-7)
Supplement: Supplementary file 1 — Supplementary file1 (DOCX 101 KB) [file 15010_2022_1826_MOESM1_ESM.docx]

# Supplementary material SARS-CoV-2 infection in chronic kidney disease patients with pre-existing dialysis: description across different pandemic intervals and effect on disease course (mortality)

Lisa Pilgram^1,2^, Lukas Eberwein^3^, Bjoern-Erik O. Jensen^4^, Carolin E.M. Jakob^5,6^, Felix C. Koehler^7,8^, Martin Hower^9^, Jan T. Kielstein^10^, Melanie Stecher^5,6^, Bernd Hohenstein^11^, Fabian Prasser^12^, Timm Westhoff^13^, Susana M. Nunes de Miranda^5^, Maria J.G.T. Vehreschild^14^, Julia Lanznaster^15*^, Sebastian Dolff^16*^, on behalf of the LEOSS study group

^1^ Department of Nephrology and Medical Intensive Care, Charité - Universitätsmedizin Berlin, Berlin, Germany.

^2^ Department of Internal Medicine, Hematology and Oncology, Goethe University Frankfurt, Frankfurt, Germany.

^3^ 4th Department of Internal Medicine, Klinikum Leverkusen gGmbH, Leverkusen, Germany.

^4^ Department of Gastroenterology, Hepatology and Infectious Diseases, Heinrich Heine University, Düsseldorf, Germany.

^5^ Department I of Internal Medicine, Faculty of Medicine and University Hospital Cologne, University of Cologne, Cologne, Germany.

^6^ German Centre for Infection Research (DZIF), partner site Bonn-Cologne, Cologne, Germany.

^7^ Department II of Internal Medicine and Center for Molecular Medicine Cologne, University of Cologne, Faculty of Medicine and University Hospital Cologne, Cologne, Germany

^8^ CECAD, University of Cologne, Faculty of Medicine and University Hospital Cologne, Cologne, Germany

^9^ Department of Pneumology, Infectiology, Internal Medicine and Intensive Care, Klinikum Dortmund GmbH, Dortmund, Hospital of University Witten / Herdecke, Germany.

^10^ Medical Clinic V, Nephrology | Rheumatology | Blood Purification, Academic Teaching Hospital Braunschweig, Braunschweig, Germany.

^11^ Nephrological Centre Villingen-Schwenningen, Villingen-Schwenningen, Germany.

^12^ Berlin Institute of Health at Charité – Universitätsmedizin Berlin, Berlin, Germany

^13^ Department of Internal Medicine I, Marien Hospital Herne Ruhr University Bochum, Herne, Germany.

^14^ Department of Internal Medicine, Infectious Diseases, Goethe University Frankfurt, Frankfurt, Germany.

^15^ Department of Internal Medicine 2, Klinikum Passau, Passau, Germany.

^16^ Department of Infectious Diseases, West German Centre of Infectious Diseases, University Hospital Essen, University Duisburg-Essen, Essen, Germany

 *contributed equally

# Supplementary figures


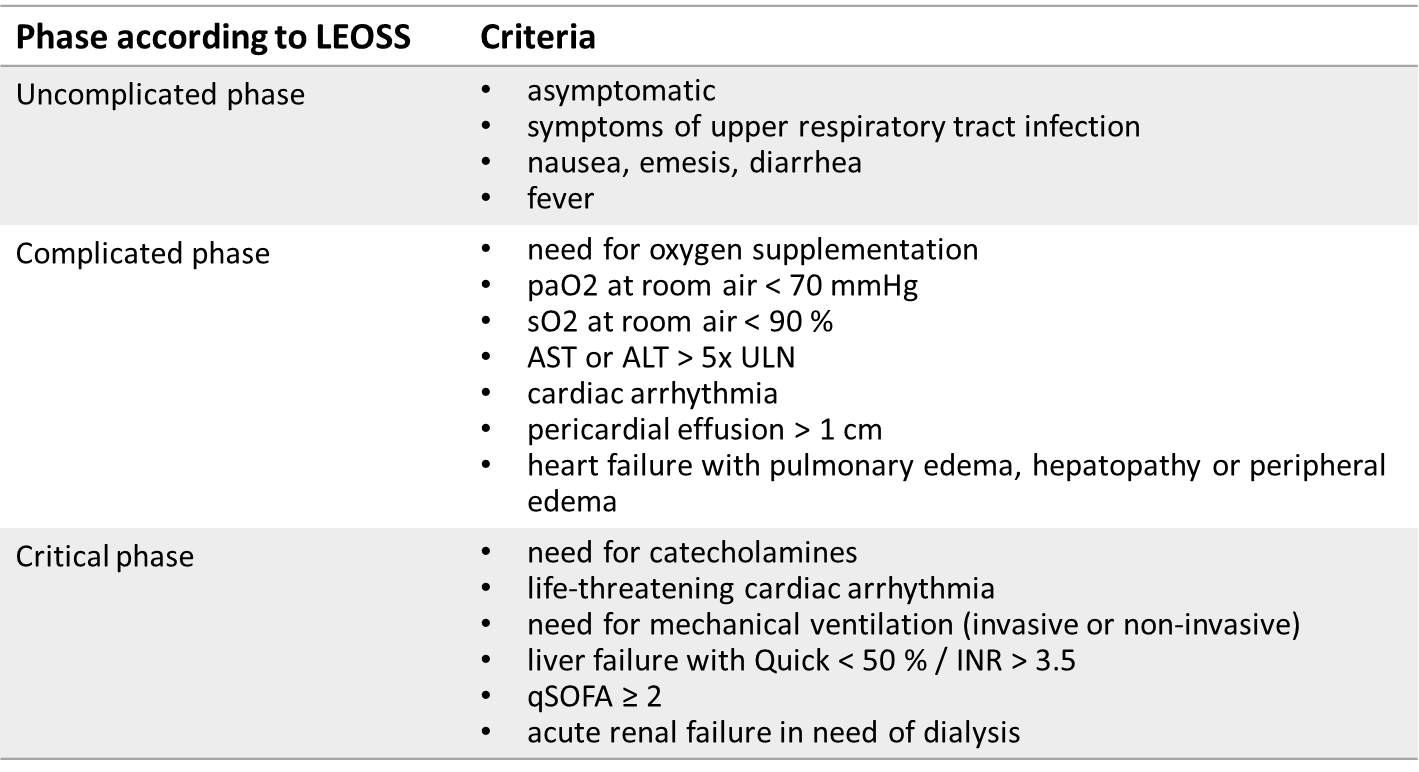


**Figure S1: LEOSS phases of COVID-19.** Meeting one criterion in the context of COVID-19 leads to an assignment to the respective phase (<https://leoss.net/statistics/>). Need for oxygen supplementation in patients with prior oxygen home therapy is defined as a clinically meaningful increase; need for mechanical ventilation in controlled ventilation (e.g. due to surgery) as a prolongation of more than 24 hours. paO2: partial pressure of oxygen. sO2: oxygen saturation. AST: aspartate aminotransferase. ALT: alanine aminotransferase. ULN: upper limit of normal. INR: international normalized ratio. qSOFA: quick sequential organ failure assessment.

# Supplementary tables

**Table S1: Missing analyses.**

| **Parameter** | **Missing values % (no.)** |
| --- | --- |
| Hypertension | 0.7 (8/1,171) |
| Chronic heart failure | 3.2 (38/1,171) |
| Coronary heart disease | 3.5 (41/1,171) |
| Diabetes mellitus type 2 | 3.8 (44/1,171) |
| Chronic obstructive pulmonary disease (COPD) | 1.8 (21/1,171) |
| Active oncological disease | 6.2 (73/1,171) |
| Obesity | 33.9 (397/1,171) |
| Pre-existing immunosuppressive therapy | 8.6 (101/1,171) |
| Steroids | 8.1 (95/1,171) |
| Remdesivir | 8.6 (101/1,171) |
| Convalescent plasma | 20.0 (234/1,171) |
| Therapy limitation | 31.6 (370/1,171) |

Missing rates are displayed for all variables containing missing values and relevant for the regression analyses.

**Table S2: Characteristics of SARS-CoV-2 infected patients suffering from CKD not requiring dialysis.**

|  | **Controls: CKD not requiring dialysis** | |
| --- | --- | --- |
|  | n=964 |  |
| 18-45 years | 35/964 | 3.6% |
| 46-55 years | 60/964 | 6.2% |
| 56-65 years | 108/964 | 11.2% |
| 66-75 years | 188/964 | 19.5% |
| 76-85 years | 368/964 | 38.2% |
| >85 years | 205/964 | 21.3% |
| **Gender** |  |  |
| Female | 396/964 | 41.1% |
| Male | 567/964 | 58.9% |
| **GFR categories (KDIGO)** |  |  |
| G1 (GFR ≥ 90 ml/min) | 67/758 | 8.9% |
| G2 (GFR 60-89 ml/min) | 114/758 | 15.0% |
| G3 (GFR 30-59 ml/min) | 395/758 | 52.1% |
| G4 (GFR 15-29 ml/min) | 120/758 | 15.8% |
| G5 (GFR < 15 ml/min) | 62/758 | 8.2% |
| **Etiology of CKD** |  |  |
| Vascular hypertensive disease | 202/441 | 45.8% |
| Secondary glomerular disease | 96/441 | 21.8% |
| Idiopathic disease | 50/441 | 11.2% |
| Primary glomerular disease | 36/441 | 8.2% |
| Polycystic kidney disease | 17/441 | 3.9% |
| Other etiologies | 40/441 | 9.1% |
| **Comorbidities** |  |  |
| Hypertension | 761/957 | 79.5% |
| Chronic heart failure | 259/933 | 27.8% |
| Coronary artery disease | 314/926 | 33.9% |
| Diabetes mellitus type 2 | 363/928 | 39.1% |
| COPD | 107/947 | 11.3% |
| Active oncological disease | 77/902 | 8.5% |
| Obesity | 196/634 | 30.9% |
| **Prior immunosuppressive medication** |  |  |
| Prior immunosuppressive medication | 187/877 | 21.3% |
| **Indicating underlying disease/status** |  |  |
| History of organ transplantation | 101/167 | 60.5% |
| Oncological disease | 32/167 | 19.2% |
| Rheumatological disease | 16/167 | 9.5% |
| Other indication | 18/167 | 10.8% |
| **Status at COVID-19 diagnosis** |  |  |
| Uncomplicated phase | 591/964 | 61.6% |
| Complicated phase | 307/964 | 32.0% |
| Critical phase | 61/964 | 6.4% |
| **Treatment in the course** |  |  |
| Steroids | 221/880 | 25.1% |
| Remdesivir | 95/879 | 10.8% |
| Convalescent plasma | 44/764 | 5.8% |
| Targeted therapy (antibodies) | 10/347 | 2.9% |
| Apheresis | 3/715 | 0.4% |
| Chloroquin | 40/879 | 4.6% |
| Azithromycin | 60/876 | 6.5% |
| **Therapy limitation** |  |  |
| Explicit deny of therapy | 172/647 | 26.6% |
| Explicit wish for therapy | 47/647 | 7.3% |
| No discussion on therapy limitations | 428/647 | 66.1% |
| **Course of disease** |  |  |
| Fatal outcome | 286/964 | 29.7% |
| Advanced respiratory support | 183/953 | 19.2% |
| Critical phase | 236/964 | 24.5% |
| Thrombotic event | 52/840 | 6.2% |
| Bleeding event | 20/775 | 2.6% |
| Septic shock | 55/960 | 5.7% |
| Congestive heart failure | 22/960 | 2.29% |

All variables are derived from the unimputed dataset and expressed as numbers (no.) and percentages (%) referred to the numbers excluding missing data (missing details in table S1). Obesity was defined by an indicated Body-Mass-Index > 30 kg/m^2^. Prior immunosuppressive medication includes an interval of 3 months before SARS-CoV-2 infection, therapy limitation defined as Do-Not-Intubate-, Do-Not-Resuscitate-Orders or the refusal of intensive care, advanced respiratory support as invasive or non-invasive mechanical ventilation or ECMO. KDIGO: Kidney Disease: Improving Global Outcomes. COPD: chronic obstructive pulmonary disease. ECMO: extracorporeal membrane oxygenation

**Table S3: Results of conditional regression analyses on fatal outcome stratified by dialysis on the unimputed dataset.**

|  | **Univariate regression analysis on fatal outcome** | | | | **Multivariable regression analysis on fatal outcome** | | | |
| --- | --- | --- | --- | --- | --- | --- | --- | --- |
|  | **OR** | **95% CI** | | **p-value** | **aOR** | **95% CI** | | **p-value** |
|  |  |  |  |  |  |  |  |  |
| Pre-existing dialysis | 0.90 | 0.42 | 1.93 | 0.781 | 1.40 | 0.73 | 2.69 | 0.313 |
| **Diagnosed between** | | | | | | | | |
| January - June 2020 | Reference |  |  |  |  |  |  |  |
| July 2020 - January 2021 | 0.40 | 0.09 | 1.82 | 0.235 | 0.80 | 0.24 | 2.72 | 0.722 |
| February - May 2021 | 0.51 | 0.96 | 1.69 | 0.424 | 0.76 | 0.18 | 3.16 | 0.707 |
| **Treatment in the course*** | | | | | | | | |
| Steroids | 0.72 | 0.23 | 2.22 | 0.561 | 0.51 | 0.18 | 1.42 | 0.195 |
| Remdesivir | 1.04 | 0.12 | 8.71 | 0.974 | 2.10 | 0.30 | 14.66 | 0.454 |
| Convalescent plasma | n.a. | n.a. | n.a. | n.a. | n.a. | n.a. | n.a. | n.a. |

Multivariable logistic regression on fatal outcome was performed after propensity-score matching using the unimputed dataset. Percentages (%) and numbers (no.) are displayed. Exact matching was performed on age, gender and phase (according to LEOSS criteria, see figure S1) at first SARS-CoV-2 detection; propensity-score matching (nearest neighbour) on hypertension, chronic heart failure, coronary artery disease, diabetes mellitus type 2, chronic obstructive pulmonary disease (COPD), active oncological disease, obesity, prior immunosuppressive medication and therapy limitations. Timing of first diagnosis was aggregated into three intervals of pandemic based on the epidemiological waves in Germany: January 2020-June 2020 (reference category), July 2020-January 2021 and February 2021-May 2021. Treatment administered at least once in the course of COVID-19 with no administration serving as reference category. Obesity was defined by an indicated Body-Mass-Index > 30 kg/m^2^. Prior immunosuppressive medication includes an interval of 3 months before SARS-CoV-2 infection. Phases at COVID-19 diagnosis were assigned according to LEOSS criteria (Figure S1). Therapy limitation were defined as Do-Not-Intubate-, Do-Not-Resuscitate-Orders or the refusal of intensive care, advanced respiratory support as invasive or non-invasive mechanical ventilation or ECMO. (a)OR: (adjusted) odds ratio. CI: confidence interval. COPD: chronic obstructive pulmonary disease. ECMO: extracorporeal membrane oxygenation. * No reference level indicated in binary variables. n.a.: excluded due to high missing rate.
